# Modeling Spinal Muscular Atrophy in Zebrafish: Current Advances and Future Perspectives

**DOI:** 10.3390/ijms25041962

**Published:** 2024-02-06

**Authors:** David Gonzalez, Constanza Vásquez-Doorman, Adolfo Luna, Miguel L. Allende

**Affiliations:** 1 Millennium Institute Center for Genome Regulation, Facultad de Ciencias, Universidad de Chile, Santiago 7800003, RM, Chile; constanza.vasquez@ubo.cl (C.V.-D.); mallende@uchile.cl (M.L.A.); 2 Departamento de Ciencias Químicas y Biológicas, Facultad de Ciencias de la Salud, Universidad Bernardo O’Higgins, Santiago 8370854, RM, Chile; adolfo.kineubo@gmail.com

**Keywords:** spinal muscular atrophy, neuromuscular junction, central nervous system, peripheral nervous system, motor neuron, neurodegenerative disease, zebrafish, *Danio rerio*

## Abstract

Spinal muscular atrophy (SMA) is an autosomal recessive neurodegenerative disease characterized by degeneration of lower motor neurons (LMNs), causing muscle weakness, atrophy, and paralysis. SMA is caused by mutations in the Survival Motor Neuron 1 (*SMN1*) gene and can be classified into four subgroups, depending on its severity. Even though the genetic component of SMA is well known, the precise mechanisms underlying its pathophysiology remain elusive. Thus far, there are three FDA-approved drugs for treating SMA. While these treatments have shown promising results, their costs are extremely high and unaffordable for most patients. Thus, more efforts are needed in order to identify novel therapeutic targets. In this context, zebrafish (*Danio rerio*) stands out as an ideal animal model for investigating neurodegenerative diseases like SMA. Its well-defined motor neuron circuits and straightforward neuromuscular structure offer distinct advantages. The zebrafish’s suitability arises from its low-cost genetic manipulation and optical transparency exhibited during larval stages, which facilitates in vivo microscopy. This review explores advancements in SMA research over the past two decades, beginning with the creation of the first zebrafish model. Our review focuses on the findings using different SMA zebrafish models generated to date, including potential therapeutic targets such as U snRNPs, Etv5b, PLS3, CORO1C, Pgrn, Cpg15, Uba1, Necdin, and Pgk1, among others. Lastly, we conclude our review by emphasizing the future perspectives in the field, namely exploiting zebrafish capacity for high-throughput screening. Zebrafish, with its unique attributes, proves to be an ideal model for studying motor neuron diseases and unraveling the complexity of neuromuscular defects.

## 1. Introduction

Spinal muscular atrophy (SMA) is an autosomal recessive neuromuscular disorder characterized by the degeneration of lower motor neurons (LMNs), ultimately leading to muscle weakness, atrophy, and paralysis [1]. In humans, it arises due to deletion or mutations in the Survival Motor Neuron 1 (*SMN1*) gene located on the 5q13 chromosome region [2]. SMN plays a pivotal role in maintaining cellular homeostasis, contributing to various essential cellular processes such as spliceosome assembly (machinery involved in removing introns from pre-mRNAs), ribonucleoprotein biogenesis (synthesis of RNA-binding proteins), mRNA trafficking, local translation, cytoskeletal dynamics, endocytosis, autophagy, bioenergetic pathways, and the ubiquitin–proteasome system [3]. Thus, *SMN1* gene mutations result in the degeneration of alpha motor neurons within the spinal cord, leading to progressive muscle weakness and eventual paralysis [4]. SMA stands out as one of the most common fatal autosomal recessive disorders, with an incidence of 1 in 10,000 to 1 in 6000 newborns worldwide [5,6], including cases in Chile. 

SMA can be classified into four groups based on the age of onset and achieved motor function: type I (0–6 months; inability to sit), type II (7–18 months; ability to sit but never stand), type III (>18 months; standing and walking during adulthood), and type IV (2^nd^-3^rd^ decade; walking unaided) [4,7]. It has been shown that within the 5q13 chromosome region, two nearly identical *SMN* genes exist: the *SMN1* gene, which is associated with SMA, and the *SMN2* gene. SMN is highly conserved and harbors multiple domains, including Gemin2-, nucleic acid- and COPI-binding domains, a Tudor domain, a proline-rich, and YG domains [8]. The distinguishing factor is a single nucleotide difference (840C>T) in the *SMN2* gene, leading to the alternative splicing of exon 7. This change produces a low amount of full-length mRNA and protein and a variable amount (~10–50%) of mRNA lacking exon 7 that is translated into a truncated SMN2 protein [9]. In this regard, over 95% of SMA patients exhibit homozygous alterations in the *SMN1* gene as a consequence of gene deletion or gene conversion to *SMN2* [4,10]. Consequently, they rely on the limited functional protein produced by the *SMN2* gene. The severity of the SMA disease is inversely related to the number of *SMN2* copies. Evidence indicates that the majority of SMA type I patients possess only two copies of SMN2 [11], while SMA type II patients have three copies, and type III–IV patients can have three to four copies [4,12,13]. 

While the genetic basis of SMA is well described, the precise mechanisms underlying the pathophysiology of this neurodegenerative disease remain elusive. Two hypotheses have been proposed to elucidate the primary impact on motor neurons. The first hypothesis suggests that the loss of SMN function in snRNP assembly may result in alterations in the splicing of specific genes. Conversely, the second hypothesis states that low levels of functional SMN disrupt mRNA transport within neurons, ultimately contributing to the development of SMA [14]. Nevertheless, accumulating evidence suggests that SMA is a multisystemic disorder affecting several organs such as skeletal muscle, heart, kidney, and spleen, among others [15]. Consequently, further studies should explore how different organs contribute to SMA progression. A comprehensive understanding of the multisystemic nature of SMA is crucial for advancing therapeutic strategies and enhancing our grasp of the complexities surrounding the condition. 

In the 1990s, the *SMN* gene was first identified and characterized as a potential causative factor for SMA [16]. Fast forward approximately two decades, the Food and Drug Administration (FDA) granted approval in 2016 for the first pharmacological treatment, nusinersen (commercially known as Spinraza^®^). Notably, infantile-onset SMA patients treated with Spinraza^®^ demonstrated remarkable enhancements in motor functions, leading to increased survival rates [17]. Furthermore, later-onset SMA patients also exhibited improved functional outcomes compared to those in the control group [18]. This significant progress shows the transformative impact of therapeutic intervention for SMA.

To date, three FDA-approved drugs are available for the treatment of SMA: Spinraza^®^, Zolgensma^®^, and Evrysdi^®^. Spinraza^®^ functions as an antisense oligonucleotide that prevents exon 7 skipping, thereby increasing the production of full-length SMN protein. This drug is administered directly to the central nervous system by injection. Similarly, Evrysdi^®^ is a small molecule that increases the amount of SMN protein by preventing exon 7 from being skipped, and it is the first oral drug developed. Zolgensma^®^ uses an adeno-associated virus as a vector to deliver a normal copy of the *SMN1* gene; the drug is administered as an intravenous infusion [19,20,21]. 

Despite the remarkable efficacy demonstrated by these drugs, their exorbitant costs render them unaffordable for the majority of individuals. Additionally, the potential side effects of these therapies remain unknown [22]. Accordingly, ethical concerns are raised about these approved drugs, especially in countries where healthcare systems restrict access to expensive therapies. It is crucial to urgently address issues about accessibility and affordability in order to ensure that individuals affected by SMA, irrespective of their socio-economic status, can avail themselves of these promising therapies. Simultaneously, efforts should be directed toward identifying novel therapeutic avenues that lead to the development of accessible treatments for patients from low-income countries. This approach is essential for widespread access to effective SMA treatments while mitigating ethical concerns related to economic disparities in healthcare access.

Zebrafish has become an attractive model for studying genetic neurodegenerative disorders, such as SMA, mostly because of its high conservation of genes involved in nervous system development and maintenance [23,24], optic transparency, lower cost and high-throughput screening possibilities compared to mouse models [1]. Although mouse models have a higher genetic similarity to humans and are widely accepted for preclinical studies, their maintenance is cost-intensive, and the experiment workflow can be time-consuming. Consequently, high-throughput drug screening using zebrafish models emerges as a potent tool, enabling the discovery of novel compounds that can subsequently be validated in SMA mouse models. Nevertheless, considering the increasing number of laboratories that are using zebrafish models for biomedical research, there is a noticeable absence of comprehensive guidelines for ensuring the welfare of these organisms. Therefore, in an effort to improve bioethical considerations, researchers should design their experiments adhering to the 3Rs principle (replacement, reduction, and refinement). This approach not only promotes ethical standards in zebrafish research but also underscores the importance of conscientious experimentation in advancing scientific knowledge.

**Figure 1:**
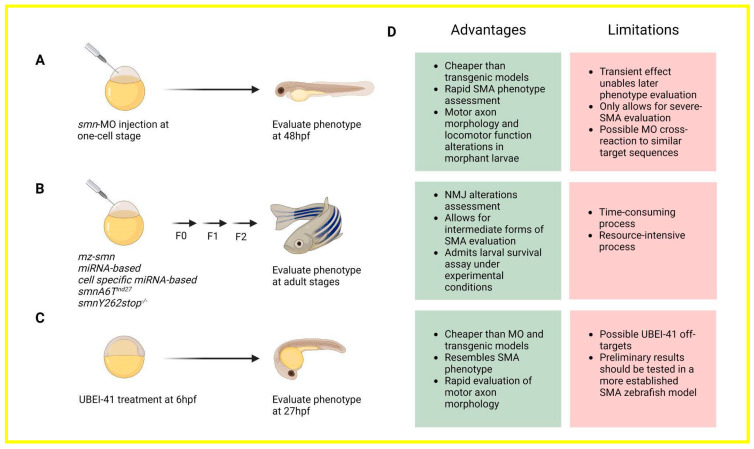
Zebrafish models employed to study SMA. To date, three different models have been utilized for studying SMA in zebrafish: (**A**) transient *smn* antisense morphant; (**B**) transgenic SMA zebrafish models; and (**C**) pharmacological UBEI-41 zebrafish model. These SMA zebrafish models have been useful to identify novel therapeutic targets and compounds, as detailed in Table 1; (**D**) advantages and limitations of each model are highlighted.

In this review, we consolidate the available evidence derived from different SMA zebrafish models, including transient Smn knockdowns achieved via antisense morpholino oligonucleotide (MO) injections, transgenic lines, and pharmacological SMA models—schematized in Figure 1. Additionally, we emphasize the discovery of novel genetic modifiers, presenting them as promising therapeutic targets for effectively treating SMA.

## 2. SMA Zebrafish Models

### 2.1. Transient smn Antisense Morphants

In 1999, Bertrandy et al. isolated for the first time *SMN* orthologous genes from both *Caenorhabditis elegans* and *Danio rerio*. Their identification revealed a domain similar to Ribonucleoprotein 1 (RNP1), a conserved octapeptide core present in RNA- and DNA-binding proteins. Therefore, the authors argued that Smn has an important role in RNA metabolism [25]. Upon the identification of Smn in zebrafish, the first attempt to model SMA disease in this organism was undertaken by McWhorter et al. in 2003. In this pioneering study, the authors found that the knockdown of Smn protein, using an MO microinjection, induced abnormalities in motor axons, even though it did not result in motor neuron death. More interestingly, they also reported that motor axon abnormalities can be rescued by wild-type Smn expression [26,27]. Another group corroborated these findings, noting that the knockdown of Smn impaired motor axon growth in zebrafish larvae [28]. 

The reduction in the Uridine-rich small nuclear ribonucleoproteins (U snRNPs) assembly pathway has been proposed as a mechanism altering transcript splicing, affecting motor neuron viability [29]. Accordingly, a study demonstrated that the knockdown of Gemin2 or pICln, two proteins involved in the U snRNPs, phenocopied SMA. Moreover, the researchers found that co-injecting *smn* MO and U snRNPs prevented motor axon alterations, indicating that degeneration of motor neurons in SMA zebrafish and patients could be a consequence of an impaired production of U snRNPs [28]. However, a few years later, McWhorter et al. presented a nuanced perspective, indicating that Gemin2 was not involved in motor axon abnormalities. The authors discussed that development and body morphologies were not initially considered when assessing motor axon alterations in an earlier study [30]. Thus, they showed that only Smn knockdown, not Gemin2, induced motor axon aberrations in zebrafish larvae. Furthermore, the researchers argued that Gemin2 exhibits distinct localization patterns and occasionally co-localizes with SMN in cultured mouse motoneurons, consistent with previous findings by others [31,32]. Based on their results, they concluded that the SMN/Gemin2 complex may not play an important role in motor axon outgrowth. However, to gain a more comprehensive understanding of the precise role of Gemin2 in SMA. In a preceding study by the same research group, it was revealed that certain mutations failed to rescue motor axon defects despite the preserved functions of snRNP, suggesting that Smn function in motor axons is independent of snRNP biogenesis [33]. 

It has been shown higher Smn protein levels in wild-type motor neurons compared to other cell types. Interestingly, this elevated transcription is attributed to the ETS variant Transcription Factor 5b (*Etv5b*). Accordingly, Etv5b knockdown results in motor axon abnormalities due to a reduction in Smn protein levels [34]. 

The actin-binding protein Plastin 3 (PLS3) has been identified as upregulated in unaffected *SMN1*-deficient patients [35]. In the same study, overexpression of human PLS3 rescued aberrant motor axons in Smn-deficient zebrafish, supporting that PLS3 acts as an SMA modifier. Furthermore, it appears that Ca^2+^ regulation is necessary for PLS3 function within motor axons since deletions of EF hands and Ca^2+^-binding sites hampered the rescue of motor axon morphology mediated by PLS3 [36]. Moreover, evidence from the same laboratory showed that overexpressing CORO1C, an F-actin binding protein, reestablished motor axon morphology in *smn*-MO zebrafish larvae [37]. Thus, the scientists hypothesized that the reduced endocytosis they observed in SMA can be reverted by PLS3 and CORO1C overexpression. In another study, the authors identified Neurocalcin delta (NCALD), a neuronal calcium sensor involved in regulating endocytosis, as a novel SMA modifier and showed that suppressing Ncald in Smn-deficient zebrafish embryos rescued motor axon abnormalities. The authors also showed that Ncald is a Ca^2+^-dependent negative regulator of endocytosis, specifically involved in vesicle recycling after neurotransmitter release [38]. More recently, they identified a novel protein called Calcineurin-like EF-hand protein 1 (CHP1), which interacts with PLS3. Using Smn-depleted zebrafish embryos, researchers found that reducing CHP1 leads to improved axonal outgrowth in motor neuron-like cells and primary SMA motor neurons [39]. Therefore, both CHP1 and Ncald suppression appear as an attractive therapeutic approach for treating SMA and other neurodegenerative diseases.

It has also been shown that over-expressing Progranulin (Pgrn), a multifunctional secreted protein, in Smn-knocked down zebrafish larvae protects motoneurons from aberrant axonal growth, suggesting that Pgrn could have a protective role against defective axonal growth in motoneurons from SMA zebrafish embryos [40]. 

Additionally, it has been shown that HuD, a neuron-specific RNA-binding protein, interacts with SMN and can also interact with several mRNAs, including candidate plasticity-related gene 15 (cpg15). Cpg15 induces motor axon branching and is also involved in neuromuscular junction (NMJ) development. The authors found that over-expressing Cpg15 in Smn-knocked down zebrafish embryos improves motor axon abnormalities, indicating that Cpg15 is a key effector of SMN and, more importantly, it could be a modifier gene of SMA disease [41]. 

In addition, it has been shown that Stasimon, a transmembrane protein involved in motor circuit function, is required for the development of motor neurons in zebrafish since the knockdown of Stasimon using a MO induces alterations in motor axons [34]. More interestingly, the investigators found that co-injection of Stasimon mRNA with *smn*-MO reverted the typical motor axon abnormalities found in Smn-deficient zebrafish but not in amyotrophic lateral sclerosis (ALS) zebrafish model, indicating that these results could be specific for the SMA zebrafish model [42]. 

A study by Gassman et al. found that small molecules known for suppressing Kinesin in *Drosophila* (SBL-154, SBL-185, and SBL-190) can rescue motor axon abnormalities from transgenic Smn-deficient and MO-injected zebrafish SMA models. However, the authors did not find an increase in the lifespan of transgenic Smn-deficient zebrafish lines when embryos were treated with SBL-154, suggesting that the effect of this compound could be restricted to motor neurons [43]. 

SMN has a role in the assembly of the spliceosome, and the Talbot group has previously shown that there are differences in splicing in SMA mice compared to controls. They also found that there are alterations in transcripts before the onset of the disease, which could potentially have a role in motor neuron integrity in SMA. One of the genes that have altered expression corresponds to *chodl*, which encodes for chondrolectin, a protein strongly expressed in motor neurons from the spinal cord in both mice and humans [44]. In this context, Talbot’s group evaluated the role of chondrolectin in an SMA zebrafish model. The authors found that co-injection of *smn*-MO and *chodl* mRNA rescued motor axon abnormalities in 28 h post-fertilization (hpf) embryos, indicating that chondrolectin is another modifier of SMA disease [45]. 

It also seems that the β-catenin signaling pathway has a detrimental role in SMA disease progression since its pharmacological inhibition using quercetin, a compound that disrupts the transcriptional activity of the β-catenin-Tcf complex, is able to partially restore motor axon morphology in *smn*-MO zebrafish larvae, suggesting that quercetin could be a novel therapeutic target for treating SMA at early stages [46]. 

Another study by Miller et al. showed that Tau phosphorylation mediates abnormalities observed in motor neurons since expressing phosphorylation-mimetic Tau forms leads to motor axon abnormalities, while non-phosphorylatable Tau forms rescue motor axon phenotype in *smn*-MO zebrafish, indicating that hyperphosphorylation of Tau, as in many neurodegenerative disorders, is also involved in SMA [47]. 

It has also been shown that UBA1, a ubiquitination enzyme downstream of ubiquitin pathways, is downregulated in SMA zebrafish. Interestingly, when *uba1* mRNA was co-injected with *smn*-MO, zebrafish larvae showed improved motor axon morphology, as well as locomotor function compared to control larvae, indicating that Uba1 is required for NMJ integrity and neuronal functions [48]. Therefore, targeting UBA1 could represent a novel therapeutic approach to treat not only SMA but also several neurodegenerative diseases. 

A different investigation showed that ATP5A, an ATP synthase mitochondrial membrane subunit, is decreased in *smn*-MO larvae, suggesting that mitochondria ATP synthesis could be compromised. Additionally, the authors showed that an important reduction in basal respiration occurred in *smn*-MO 24 hpf larvae [49]. More importantly, overexpression of Necdin, a protein involved in neuronal mitochondria biogenesis, rescued motor axon abnormalities in *smn*-MO larvae. Even more, the investigators evaluated the role of Phosphoglycerate kinase 1 (PGK1), a protein involved in ATP synthesis in the glycolytic process and found that overexpression or pharmacological induction of PGK1 also rescues motor axon abnormalities in *smn*-MO zebrafish larvae, indicating that these pathways are potential therapeutic targets for treating SMA disease [49]. 

An additional study showed that Smn dynamics are composed of two components, a fast and a slow component. They showed that slow components might likely represent Smn oligomers, while fast components represent Smn monomers. In this regard, the authors showed that knocking down endogenous Smn alters differently its dynamics in cell bodies and axons of the SMA zebrafish model, in which they found that there is a depletion of exogenous monomeric Smn in cell bodies, meaning that only the slow component is observable. While in axons, the researchers observed a reduction in diffusion of both fast and slow components. Interestingly, dynamics of truncated-Smn (eGFP-SmnΔex6,7) failed to form oligomers, and it was found to diffuse in both soma and axons, while the slow components were faster compared to slow components in the presence of full-length eGFP-Smn [50]. Thus, the authors suggest that slow components could be composed of protein complexes that bind to Smn, while diffusional particles could be Smn associated with messenger RNP complexes. 

Lastly, the Smn-deficient SMA zebrafish model has also been used to study the role of sumoylation in Smn localization and integrity. In this context, it has been shown that inactivating the SUMO-interacting motif (SIM) of Smn protein not only alters Smn intracellular localization but also fails to rescue motor axon abnormalities in the SMA zebrafish model, indicating that interactions between SUMO and SIM are key for Smn function during development of motor neurons in zebrafish [51].

Taking all together, the transient knockdown of Smn in zebrafish recapitulates many aspects of SMA disease and allows for the identification of novel SMA modifiers that could be useful as therapeutic targets.

### 2.2. SMA Transgenic Zebrafish Models

The generation of the first genetic spinal muscular atrophy zebrafish model was performed by Boon et al. in 2009. In this study, the authors showed that mutant SMA zebrafish (*smnY262stop*, *smnL265stop*, *smnG264D*) were smaller and died young during the second (*smnY262stop* and *smnL265stop*) or third week (*smnG264D*) of age. The mutant SMA zebrafish also exhibited a decrease in synaptic vesicle protein (SV2), although two other SV proteins were unaffected (synaptotagmin and synaptophysin). More interestingly, they found that motor neurons require Smn to maintain SV2 in the presynaptic terminals, suggesting that Smn could play an important role at this site [52]. Additionally, it was shown that motor neuron-specific expression of SMN in the *mz-smn* transgenic zebrafish line, which lacks both maternal and zygotic Smn and expresses low levels of human RFP-SMN, rescues abnormalities in the development of motor neurons; even more, the authors found that it also rescues aberrant development of Schwann cells (SCs), and short peripheral axon abnormalities in neurons from the dorsal root ganglia (DRG). However, they did not find an increase in zebrafish lifespan, which could suggest that lack of SMN in other cell types may contribute to animal survival [53]. 

A novel miRNA-based approach has been developed to generate models to study human diseases in zebrafish [54]. This method consists of knocking down a specific gene by using miRNA-expressing constructs. Thus, the authors generated a *smn1* knockdown transgenic line that recapitulates SMA phenotypes. They found that the severity of the SMA phenotype correlates with the level of *smn1* inhibition [54]. Thus, this tool could also be useful for other gene knockouts that are lethal at embryonic stages. Using this approach, Laird et al. generated a cell type-specific smn1 silencing strategy to downregulate Smn1 either in motor neurons or skeletal muscle [55]. The investigators found that the reduction in Smn1 protein levels in motor neurons recapitulates SMA phenotypes. Interestingly, they did not find SMA signs in muscle-specific *smn1* downregulated larvae, demonstrating that *smn1* downregulation in motor neurons is sufficient to cause SMA phenotypes in zebrafish [55]. 

More recently, the laboratory of Dr. Winkler has generated the *smnA6T^ind27^* zebrafish model for an intermediate type of SMA, which has normal Smn levels during early stages and decreased Smn levels in later juveniles. In this model, both motor neurons and NMJs initially develop normally, but a reduction in motor neuron number and alteration in NMJ integrity are observed at 36 hpf. Additionally, skeletal muscle architecture is also affected at this stage, in which myofibers with the smallest cross-sectional area are more abundant. As expected, the authors found that the *smnA6T^ind27^* zebrafish line showed defects in locomotor function, indicating that Smn is important not only for motor neuron and NMJ development but also for their maintenance [56]. On the other hand, it seems that HuD, an RNA binding protein, is also important for motor neuron development since HuD mutant zebrafish lines show a reduction in motor axon complexity accompanied by reduced locomotor function. Interestingly, motor axon abnormalities were rescued when HuD was expressed in Smn-depleted zebrafish larvae. Additionally, the researchers found that Gap43, a protein involved in axonal outgrowth, is decreased in Smn-depleted zebrafish larvae, and expressing HuD in Smn- and HuD-depleted zebrafish lines reestablishes Gap43 levels [57]. Thus, the authors hypothesize that decreased levels of HuD result in a perturbed mRNA transport into the axons, affecting axonal development, while higher levels of HuD in Smn-depleted embryos can compensate for low levels of Smn [57].

Another study by Hao et al. showed a novel genetic zebrafish model for studying SMA disease. They have generated a transgenic zebrafish line that expresses low amounts of the human *SMN2* gene (*hSMN2*) in an *smn*^−/−^ background. They also showed that antisense oligonucleotide treatment is able to restore *hSMN2* splicing, increasing the full-length *hSMN* mRNA. Furthermore, the hSMN2 protein is able to rescue the SMA phenotype and also increase the survival of *smn^−/−^* mutant zebrafish larvae. Thus, the authors propose this model as a novel tool for developing drug screening and approach that increases the length of the SMN protein [58]. A different investigation from the same group showed that SMN protein affects PLS3, a modifier gene of the SMA disease, which leads to motor deficiencies [59], supporting the results found by MO injections.

### 2.3. Pharmacological SMA Zebrafish Model

A pharmacological model of SMA was used to test small-molecule compounds in zebrafish. This particular model consists of inhibiting the ubiquitin-like modifier activating enzyme 1 (Uba1), which binds to Smn, by using the UBEI-41 inhibitor in wild-type zebrafish larvae [46]. Notably, UBA1 has been found to be reduced in SMA mice [46]. Its relevance in the SMA context is underscored by mutations in exon 15 of the human *UBE1* gene, responsible for encoding UBA1 (also known as UBE1). These mutations lead to decreased UBA1 levels, giving rise to X-linked SMA—a distinct SMA form similar to type I, which exclusively affects males [60]. Given the connection between UBA1 and SMA pathology, disruptions in the UBE1-mediated ubiquitin–proteasome system could potentially impede motor neuron development, contributing to X-linked SMA. The use of the UBEI-41 inhibitor presents an appealing pharmacological approach to model X-linked SMA in zebrafish. In this regard, the inhibition of Uba1 leads to defects in motor axons, similar to SMA zebrafish models. Thus, by performing an in vivo drug screening in zebrafish embryos, the authors identified a drug called dipyridamole, an adenosine uptake inhibitor, that partially rescues motor axon architecture in UBEI-41-treated zebrafish embryos [61]. The authors explored the potential therapeutic effects of dipyridamole, highlighting its dual action in blocking adenosine uptake and inhibiting phosphodiesterases. They suggested that these actions could contribute to the stability of the NMJ. Moreover, dipyridamole has demonstrated antioxidant properties with neuroprotective effects in CNS neurons in vitro [62]. Interestingly, it also exhibited neuroprotective effects in α-synuclein-induced neurotoxicity, a mechanism implicated in Parkinson’s disease [63]. This body of evidence shows a promising therapeutic potential for dipyridamole not only for treating SMA but also for other neurodegenerative diseases involving NMJ alterations or α-synuclein-induced neurotoxicity.

Even though a few studies have used a pharmacological approach to study SMA, its potential seems to be exceptional and is allowing researchers to perform high-throughput drug screening to identify small-molecule compounds among hundreds of potential candidates, such as dipyridamole. Nevertheless, additional studies are needed to discern possible UBEI-41 off-targets and/or other alterations triggered by Uba1 inhibition in zebrafish larvae. In this context, the pharmacological UBEI-41 model for SMA proves valuable in pinpointing potential therapeutic targets or compounds. The insights gained from this model can be tested in other SMA animal models to further validate its beneficial effects. The comprehensive exploration of its effects and interactions will contribute to refining and optimizing therapeutic strategies for SMA treatment. 

## 3. Concluding Remarks and Future Perspectives

As reviewed in this article, zebrafish is emerging as an outstanding animal model to study neurodegenerative diseases, mainly because of its simple and low-cost genetic manipulation, allowing researchers to model a variety of genetic diseases either to test novel molecules with therapeutic potential, or to identify genetic modifiers that could potentially alleviate disease progression. Zebrafish also have a relatively similar nervous system organization compared to mammals and possess LMNs, which are affected in by SMA. They also have a similar excitation–-contraction coupling machinery and contractile apparatus compared to humans [1]. Additionally, they offer optic transparency at larval stages, which is very suitable when performing in vivo microscopy, and also, SMA phenotypes are easily distinguishable during the first daysday’s post-fertilization.

Regarding SMA, zebrafish models have been useful to explore the contribution of several proteins to the disease’s progression, summarized in Table 1 and compared in Figure 1. To date, researchers have been using three different approaches: Smn knock-down by MO injection, transgenic, and pharmacological models (by pharmacologically suppressing UBA1). Most of the studies have used the transient MO-mediated Smn knock-down model, probably due to its simplicity, low-costlow cost, and time savingtime-saving features that allow a rapid and measurable SMA phenotype, such as alteration in axon morphologies and locomotor functions. These studies have successfully identified numerous targets capable of reversing the SMA phenotype. Notable targets include U snRNPs, Etv5b, PLS3, CORO1C, Pgrn, Cpg15, Stasimon, non-P Tau, Uba1, Necdin, and Pgk1. On the other hand, the use of transgenic SMA zebrafish models has enabled researchers to assess later-stage SMA phenotypes, replicating milder forms of the condition. This model allows for the specific downregulation of *smn* in particular cell types and has facilitated the identification of targets that can rescue SMA phenotypes, such as HuD, PLS3, and hSMN2. Lastly, only a few studies have used a pharmacological approach to study SMA based on inhibiting UBA1 [46,61]; nevertheless, exceptional findings have been made using this approach, and it allows researchers to identify novel drug candidates via high-throughput drug screening in zebrafish larvae. While SMA zebrafish models provide outstanding insights, it is crucial to acknowledge certain limitations. For instance, there are some variations in the complexity of the organization of the CNS, peripheral nervous system, and NMJ architecture. Additionally, the zebrafish possesses overall limitations, such as small size, rapid development, and short lifespan in comparison to humans. Regardless of these limitations, the translational potential of SMA zebrafish models is valuable.

**Table 1:** Summary of findings using different SMA zebrafish models.

Protein/Gene/Molecule	Function	Experimental Condition	Results	Reference(s)
Transient SMA antisense morpholino models				
Smn	Deletion or mutations in Smn are linked to SMA	Knocked down	Motor axon abnormalities	[26]
U snRNP	Small nuclear ribonucleoproteins involved in forming spliceosome	Knocked down	Rescues Smn-deficient zebrafish	[28]
Etv5b	Transcription factor orthologous to human ETV5	Knocked down	Phenocopies Smn-deficient zebrafish	[34]
PLS3	Actin-binding protein	Overexpressed	Rescues Smn-deficient zebrafish	[35,36]
CORO1C	F-actin-binding protein	Overexpressed	Rescues Smn-deficient zebrafish	[37]
Pgrn	Secreted glycoprotein involved in several cell processes	Knocked down	Rescues Smn-deficient zebrafish	[40]
Cpg15	Promotes axon branching and NMJ formation; highly expressed in developing ventral spinal cord	Overexpressed	Rescues Smn-deficient zebrafish	[41]
Stasimon	Transmembrane protein involved in motor circuit function	Knocked down	Phenocopies Smn-deficient zebrafish	[42]
SBL-154, SBL-185, SBL-190	Small molecules that lower Aβ42 production	-	Rescues Smn-deficient zebrafish	[43]
Quercetin	Disrupts activity of the β-catenin-Tcf complex	Pharmacologically inhibited	Rescues Smn-deficient zebrafish	[45]
Non-P tau	Microtubule-assembly factor, regulator of intracellular trafficking	Overexpressed	Rescues Smn-deficient zebrafish	[47]
Uba1	Ubiquitination enzyme downstream ubiquitin pathways	Overexpressed	Rescues Smn-deficient zebrafish	[48]
Necdin	Involved in neuronal mitochondria biogenesis	Overexpressed	Rescues Smn-deficient zebrafish	[49]
Pgk1	Involved in ATP synthesis in the glycolytic process	Overexpressed/ Pharmacological induction	Rescues Smn-deficient zebrafish	[49]
Transgenic SMA zebrafish models				
*smn*	-	*smnY262stop*, *smnL265stop*, *smnG264D* mutations	Decreased levels of SV2	[52]
*smn*	-	*mz-smn*	Abnormal SC, DRG, and motor neuron development	[53]
*smn*	-	miRNA-based	Recapitulates different forms of SMA	[54]
*smn*	-	Cell-specific miRNA-based	*smn1* downregulation in motor neurons causes SMA	[55]
*smn*	-	*smnA6T^ind27^*	Affected motor neurons, NMJ, and skeletal muscles in late stages	[56]
HuD	RNA-binding protein expressed early in motor neurons	Overexpressed in *mz-smn*	Rescues motor axon and locomotor alterations	[57]
PLS3	Actin-binding protein	Overexpressed in *smn^−/−^* (*smnY262stop*)	Rescues locomotor function	[59]
hSMN2	Human SMN2	Overexpressed in *smn^−/−^* (*smnY262stop*)	Rescues low levels of SV2 observed in *smn^−/−^* larvae	[58]
Pharmacological SMN zebrafish models				
Uba1	Ubiquitination enzyme downstream ubiquitin pathways	Uba1 inhibition (UBEI-41)	Motor axon alterations	[46]
Dipyridamole	Adenosine uptake inhibitor	Treated Uba1-inhibited (UBEI-41)	Rescues motor axon abnormalities	[61]

Despite the substantial progress made in unraveling the pathophysiology of SMA, there is a critical gap in understanding how SMN deficiency leads to the degeneration of motor neurons. However, SMA is beginning to be considered a multisystemic disorder that extends beyond the CNS. Consequently, it is imperative for researchers to address how SMN deficiency impacts different organs and how it contributes to SMA progression. Furthermore, exploring the roles of different glial cells within both the CNS and the peripheral nervous system becomes crucial in gaining a holistic understanding of SMA pathology.

Thus, it is plausible to think that in the coming years, zebrafish will continue to attract the attention of researchers, and further studies will shed light on the mechanisms underlying SMA pathophysiology, as well as it will continue to be useful to identify new targets with therapeutic potential. As mentioned above, even though there are three FDA-approved drugs for SMA, they are extremely expensive and unaffordable for most people. Because of this scenario, we believe that exploiting the capacity of zebrafish to perform high-throughput drug screening will be crucial to further identify compounds that can slow down SMA disease progression at a lower cost. Furthermore, other powerful approaches, such as drug repurposing, might provide insights into potential therapeutic targets for neurodegenerative diseases using existing drugs. However, researchers need to be cautious about translatability to humans since differences in drug metabolism might exist. Therefore, it is mandatory to continue investigating SMA etiology in order to find novel therapeutic avenues to ultimately improve the quality of life of patients.

## Data Availability

No new data were created in this review article.
